# The role of pyroptosis and its crosstalk with immune therapy in breast cancer

**DOI:** 10.3389/fimmu.2022.973935

**Published:** 2022-08-30

**Authors:** Ling Wu, Hongsheng Lu, Yin Pan, Chen Liu, Jinyan Wang, Baofu Chen, Yichao Wang

**Affiliations:** ^1^ Department of Clinical Laboratory Medicine, Taizhou Central Hospital (Taizhou Univesity Hospital), Taizhou, China; ^2^ Medical College, Yangzhou University, Yangzhou, China

**Keywords:** breast cancer, pyroptosis, gasdermin, inflammasomes, immunotherapy

## Abstract

Pyroptosis is a brand-new category of programmed cell death (PCD) that is brought on by multitudinous inflammasomes, which can recognize several stimuli to pilot the cleavage of and activate inflammatory cytokines like IL-18 and IL-1β is believed to have dual effects on the development of multiple cancers including breast cancer. However, pyroptosis has different effects on cancers depending on the type of tissues and their distinct heredity. Recently, the association between pyroptosis and breast cancer has received more and more attention, and it is thought that inducing pyroptosis could be used as a cancer treatment option. In addition, a great deal of evidence accumulating over the past decades has evinced the crosstalk between pyroptosis and tumor immunological therapy. Thus, a comprehensive summary combining the function of pyroptosis in breast cancer and antitumor immunity is imperative. We portray the prevalent knowledge of the multidimensional roles of pyroptosis in cancer and summarize the pyroptosis in breast cancer principally. Moreover, we elucidate the influence of inflammasomes and pyroptosis-produced cytokines on the tumor microenvironment (TME) of breast cancer. Taken together, we aim to provide a clue to harness pyroptosis rationally and apply it to augment immunotherapy efficiency for breast cancer.

## Introduction

The Global Cancer Statistics 2020 showed that breast cancer is now the most prevalent worldwide cancer, constituting 11.7% of the anticipated 2.3 million global cancer cases, and being the first cause of cancer death among women (1). From a morphological, phenotypic, and molecular perspective, breast cancer is very heterogeneous (2). Its histological classification is primarily grounded on the expression or occurrence of hormone receptors including human epidermal growth factor receptors 2 (HER2) and estrogen receptors (ER), progesterone receptors (PR), and the proliferation marker Ki-67. There are four main categories of breast cancer according to the presence/absence of molecular markers mentioned above: HER2+ (ER−, PR−, HER2+), luminal A (ER+ and/or PR+, HER2−, Ki-67<14%), luminal B (ER+ and/or PR+, HER2+ or HER2−, Ki-67 >14%) and triple-negative breast cancer (TNBC) (ER−, PR−, HER2−) (3). About 10% of breast cancer patients grow into metastatic disease and 90% are not metastatic when the diagnosis was made. The Therapeutic measures of breast cancer are classified into systemic therapy and local therapy based on the subtype and the degree of metastasis (4). For diagnosed breast cancer patients present with nonmetastatic, the therapies are aimed at eradicating the full extent of the tumor and preventing tumor metastasis and recidivism. Local therapy includes surgery and postoperative radiation while neoadjuvant (preoperative) and adjuvant (postoperative) treatment are defined as systemic therapy. The standard systemic therapy is dependent on the breast cancer subtype, including chemotherapy alone for TNBC, endocrine therapy which is the cornerstone of HER2−/ER+/PR+ breast cancer, and immunotherapy (i.e., trastuzumab and pertuzumab) for HER2+ breast cancer. Currently, almost all patients present with metastatic breast cancer remain incurable virtually, local therapy together with systemic therapy is normally prescribed to the therapeutic purpose of relieving the symptoms and extending life (5). Although radical surgical resection is the most curative therapy, patients diagnosed with later-stage breast tumors face a significant risk of morbidity and death rates, which poses an enormous challenge for the prognosis and therapeutic effect of breast cancer. In addition, the prognosis of patients with advanced-stage tumors remains abysmal because of the resistance to radiotherapy and chemotherapy.

The paradigm of treatment has shifted from standardized treatment regimens to “precision medicine” as a result of advancements in breast cancer diagnosis and management, which targets the particular genetic composition of the tumor (6). For that reason, the deep insight into the potential molecular mechanism of breast cancer therapy may serve as a precondition to develop new treatment strategies. It is urgent to explore additional clinical approaches for effective breast cancer intervention and increase these tumors’ sensitivity to chemotherapy and radiotherapy. Emerging evidence has revealed that pyroptosis has positive significance for developing new multiple malignant tumors including breast cancer therapy regimens, degrading the tolerance to chemotherapy, and prolonging patients’ life span. Pyroptosis, a type of PCD relating to or causing lysis and inflammation, is actuated by diverse incitements. And extensive research has been carried out on proptosis and various diseases. Since the advent of the protein known as gasdermin D (GSDMD), which is engaged in the pyroptotic process in 2015, abundant treatments have been developed to trigger pyroptosis for cancer. Pyroptosis also has a close correlation with the transition of immunity in the TME, which can use the immune response to mediate pyroptosis and treat cancer (7). Due to its hypoergia in the lymphocytic infiltrate, cancer mutational load, and responsiveness to anti-programmed death-1/programmed death ligand-1 (PD-1/PD-L1) treatment, breast cancer was historically thought to be immunologically “cool” and “quiet”. However, in the last few decades, with the evolutionary discoveries on immune checkpoints and breakthroughs of molecular biology, immunotherapy has achieved remarkable success in breast cancer treatments (8). And a substantial amount of evidence has demonstrated that effective monotherapy necessitates sustained anti-cancer immunity to lower tumor recurrence. Despite the fact that pyroptosis has drawn more attention because of its conflicting effects on immunotherapy and cancer, a less elaborated summary covered the functions of pyroptosis and the close connection between the innate immune system and pyroptosis-mediated treatment in breast cancer has been reported. Hence, it is compulsory to fill this vacancy and provide guidelines for our future directions to make use of such a mighty implement in the fight against breast cancer and the treatment option.

This review covers an introduction to pyroptosis and a summary of all the current knowledge about its roles in the tumorgenesis and development of breast cancer, as well as its molecular mechanism in breast cancer. Through a systematic search based on four common databases, the current pyroptosis mechanisms of breast cancer mediated by gasdermins, the main executors of pyroptosis, will be described in detail. We also concentrate on how pyroptosis regulates tumor immunity. The possible roles of pyroptosis-related inflammasomes, gasdermins, and cytokines like interleukin-1β (IL-1β) and interleukin-18 (IL-18) in the TME of breast cancer are further elucidated to explore potential targets contributing to fight against breast cancer. With this review, we attempted to broaden our understanding of pyroptosis and how it interacts with immune treatment for breast cancer, which will provide an avenue for illuminating more actionable targets and candidate drugs for potential pyroptosis-related breast cancer therapy.

## Overview of pyroptosis

In a multicellular organism, it is extremely important to keep the proper equilibrium between cell survival and mortality in the physiological process and disease course. Among those processes, PCD plays a significant role in homeostasis (9). PCD refers to the tightly regulated, genetically controlled, self-orchestrated processes of cell death relying on certain genes that encode signals. Based on considerable research on biochemical features and intricate mechanisms, multiple PCD forms have been found containing apoptosis, autophagy, necroptosis, ferroptosis, pyroptosis, PANoptosis, etc (10). Pyroptosis is an inflammatory PCD mediated by inflammasomes which can cleave the gasdermin family proteins and awaken the inactive cytokines like IL-1β and IL-18 (11). Pyroptosis differs from other forms of PCD in morphology and biochemistry because it finally leads to the creation of cell membrane perforations, the loss of ion homeostasis, the release of inflammatory mediators, and the emergence of huge bubbles from the plasma membrane (12).

**Figure 1:**
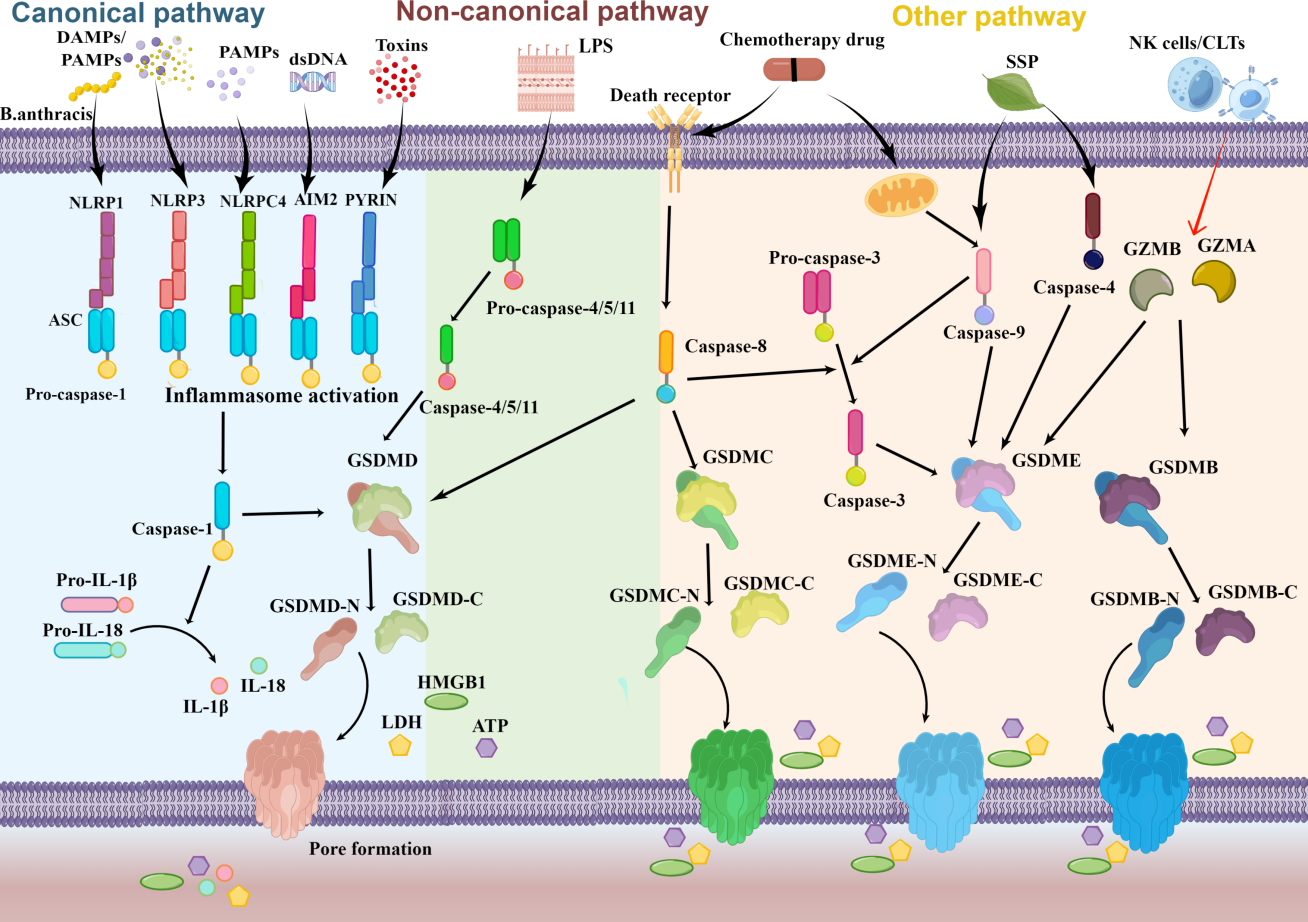
Schematic molecular mechanisms representation of pyroptosis pathway. Numerous factors such as DAMPs and PAMPs trigger the canonical inflammasome pyroptosis pathway to occur and activate pro-caspase-1. Cytoplasmic sensors of those stimuli include NLRs, AIM2, and Pyrin. Pro-IL-1β and pro-IL-18 then mature as a result of activated caspase-1. In the non-canonical pathway, activated caspase-11/4/5 is facilitated by direct recognition of intracellular LPS. Activated caspase-1/11/4/5 release the N-terminal domain of GSDMD (GSDMD-N) from the auto-inhibitory C-terminal domain of GSDMD (GSDMD-C) by cleaving full GSDMD. When GSDMD-N reaches the plasma membrane, it oligomerizes and creates a pore that ultimately aids in cell lysis. The formation of pore supports the secretion of intracellular content (HMGB1, LDH, ATP) and the inflammatory cytokines IL-18 and IL-1β activated by caspase-1. Caspase-8 may additionally cleave GSDMD and GSDMC through other pyroptosis pathways. Besides it, caspase-3/4/9, granzyme B (GZMB), and granzyme A (GZMA) may all act on GSDME and GSDMB, respectively.

Pyroptosis is firstly discovered in rapid macrophage death induced by Shigella flexneri in 1992 and was regarded as apoptosis at first because of the similar characteristics (13). However, further studies showed this induction of macrophage apoptosis resulted in a considerable release of IL-1, specifically IL-1β, and only modest levels of IL-6 and TNF-α (14). Subsequent studies indicated that the biochemistry of this apoptosis induced by *Salmonella* had a remarkable resemblance to necrosis. At the same time, the caspase-1 was activated, which clearly distinguished it from other kinds of cell deaths. Beyond that, the membranes of apoptosis cells were complete while the integrity of the infected macrophages cell membrane was ruined (15, 16). Those distinctions above showed that the macrophage death induced by *Salmonella* was different from its apoptotic counterpart. Thus, in 2001, Boise et al. (17) embraced the concept that death induced by *Salmonella* was programmed as discussed by Boise and Collins and coined the appellation pyroptosis to describe it. The terminology “pyroptosis” derives from the Greek terms “pyro,” which refers to fire or fever, and “ptosis,” which means “falling” (18). From then on, though there have been a plethora of reports related to pyroptosis, the upstream and downstream mechanisms of caspase-1 activation were still unclear. In 2002, Fabio Martinon (19) firstly reported the identification of a compound called inflammasome that activates caspase, which was a breakthrough in the understanding of the activation of the caspase-1. Since then, various inflammasomes composed of different receptors of caspase-1 activation have been identified. Afterward, in 2015, Shao et al. firstly discovered that GSDMD was a target cleavaged by caspase-1 (20) (**Figure 1**), which uncovered the downstream mechanisms of pyroptosis. In addition to this, more and more pieces of evidence have shown that pyroptosis is also brought on by gasdermin E (GSDME) triggered by caspase-3 (21) (**Figure 1**). So far, there are six categories of human gasdermins classified based on the difference in the conserved domain: gasdermin A (GSDMA), gasdermin B (GSDMB), gasdermin C (GSDMC), GSDMD, GSDME/DFNA5 (deafness, autosomal dominant 5)), and DFNB59/Pejvakin) (22). While mice possess ten gasdermins including three homologs GSDMA (GSDMA1-3), four homologs GSDMC (GSDMC1-4), and one homolog each of GSDMD, GSDME, and DFNB59. Except for DFNB59, the gasdermins proteins are conserved in the N-terminal domain and involved in certain biological functions, especially in forming plasma membrane pores and inducing pyroptosis (23, 24).

Experiments carried out on mice and human cells have repeatedly demonstrated that pyroptosis is induced by different caspases. According to the study reported by Kayagaki et al. (25), there are two distinct pyroptosis pathways: the canonical pathway which leads to the activation of inflammasome caspase-1, and the non-canonical pathway, which involves the activation of inflammasome caspase-11. And Subsequent studies have shown that the orthologs of mouse caspase-11, caspase-4, and caspase-5 function on noncanonical pathways in human cells (26–28) (**Figure 1**). Inflammasomes serve as the molecular platform to activate caspase-1-mediated inflammatory responses, which promote the secretion of bioactive IL-1β and IL-18 and induce pyroptosis ultimately. Sensor molecules, typically pattern recognition receptors (PRRs), and adaptor apoptosis-associated speck-like protein (ASC) are the building blocks of inflammasomes (29). In the canonical inflammasome pathway, the recruitment of ASC and pro-caspases to form inflammasomes occurs as a result of PRRs recognizing damage- or pathogen-associated molecular patterns (DAMPs or PAMPs). PRRs related to pyroptosis mainly encompassed three gene families: intracellular nucleotide-binding oligomerization domain (NOD)-like receptors (NLRs), absent in melanoma 2 (AIM2)-like receptors (ALRs), and Pyrin (30). Depending on the type, PRRs can recognize different DAMPs or PAMPs. NLR inflammasomes can be initiated by adenosine triphosphoric acid, bacterial toxins, exogenous pathogens, and endogenous damage signals (31) while AIM2 mainly responds to cytosolic DNA stimulation during bacterial or viral infection (32). PRRs comprise either leucine-rich repeats (LRR) or DNA binding HIN-200 domain in the C-terminal sever as ligand recognition and autoinhibition and caspase recruitment (CARD) or pyrin (PYD) domains in the N-terminal that control the signaling event (33). A central nucleotide-binding oligomerization domain (NOD/NACHT) domain is also in NLR proteins and utilizes ATP to activate the signaling complex (31). After the C-terminal domain recognizes the stimuli from the corresponding DAMPs or PAMPs, the conformation of PRRs changes to relieve self-inhibition. And the ASC is made up of PYD and CARD docks onto the PRRs hub *via* PYD-PYD interactions and then recruits pro-caspase-1 employing CARD-CARD interactions to create the typical inflammasome (34). The pro-caspase‐1 recruited to the inflammasome is activated to form caspase‐1 which catalytically slices GSDMD in the middle linker to separate the domain of N-terminal (GSDMD-N) and C-terminal (GSDMD-C) rapidly, removing the inhibitable action on the GSDMD-N. In the plasma membrane, GSDMD-N then bind to the phosphoinositide in the cell membrane to generate oligomeric holes (20), leading to elevated membrane permeability, swelling, bubble formation, and the ultimate plasma membrane rupture to release high mobility group protein (HMGB1), lactate dehydrogenase (LDH, and)ATP (35–37). Besides it, caspase-1 has the ability to cut the precursor protein of IL-1β and IL-18 and maturate those molecules, which are then released into the nearby immunological system and eventually stimulate the complete immune response to eliminate invading pathogens. Meanwhile, mature IL-1 and IL-18 can be secreted through membrane pores created by GSDMD. In conclusion, caspase‐1‐dependent pyroptosis mediated by inflammasome assembly constitutes the classical canonical pathway.

Activated caspase-4/5/11 triggers pyroptosis specifically connect with the lipid A through binding bacterial lipopolysaccharide (LPS) in the non-canonical pathway, which triggers caspase-4/5/11 oligomerization and activation to autonomously cleaves the GSDMD, eventually leading to pyroptosis (38). Caspase-4/5 does not mature proproteins IL-1β and IL-18, but caspase-11 does promote a modest amount of IL-1β release dependent on the mediation of NLRP3 inflammasome (25). The release of ATP induced by caspase-11 activates an ion channel, resulting in cell rupture and potassium (K+) efflux which in turn triggers NLRP3 inflammasome and inevitably stimulated the release of IL-1β (39, 40).

Pyroptosis can also be initiated by certain additional caspases, including caspase-3 (21) and caspase-8 (41) as well as caspase-9 (42) (**Figure 1**). Using either caspase-8 or caspase-9, the death receptor-mediated or the mitochondrial apoptotic pathway activates caspase-3 (43). With the treatment of chemotherapy drugs or viral infection, caspase-3 selectively cleavages GSDME to release the GSDME-N, which subsequently stimulates the activation of the pyroptosis (21, 44). Zhang et al. (42) also uncovered that Spatholobus suberctus Dunn percolation extract (SSP) elevated caspase-4 and caspase-9 to cleave GSDME, inducing the permeabilization of the cell membrane and pyroptosis. Moreover, antibiotic chemotherapy drugs can induce pyroptosis in a GSDMC manner mediated by caspase-8 (45). And recent studies also indicated that cytosolic caspase-8 was capable of cleaving GSDMD to induce pyroptosis when it was activated by ligands of Toll-like receptors 3 and 4 (TLR3 and TLR4) or tumor necrosis factor (TNF) (41, 46, 47). More unexpected is that pyroptosis can also be activated by granzymes (GZMs) released by natural killer (NK)cells and cytoplasmic granules within cytotoxic T cells (CTLs) (**Figure 1**). Liu et al. (48) initially stated that Granzyme B (GZMB) liberated from chimeric antigen receptor T cells allowed them to briefly stimulate caspase-3 in leukemic cells and cause GSDME-dependent pyroptosis in 2020. Furthermore, studies identified that GZMB can straightway incise GSDME to trigger pyroptosis (49). Additionally, it has been demonstrated that the lymphocyte-derived granzyme A (GZMA) hydrolyzes GSDMB at Lys229/Lys244 site to cause tumor cell pyroptosis in the same year (50).

## The roles of pyroptosis in cancer

Cancer cells are characteristic of unlimited proliferation and organisms try to take advantage of the regulation of the protective mechanism of normal cells to restrict cell proliferation and suppress tumor development in normal physiological conditions (51). However, cancer cells have numerous tactics to escape or curb the cell death process that mediates the natural cell death process. The daedal molecular management of the internet signal-mediated death process is known to participate in the initiation, proliferation, metastasis, and even treatment effect of malignant cells. Thus, stimulation of cellular demise may be a possible therapeutic target for cancers. Emerging shreds of evidence have shown that pyroptosis occurs in various malignant cells. As an inflammatory cell death, the crucial elements in the pyroptosis such as inflammasomes, gasdermin proteins, and inflammatory cytokines, take part in the transformation and development of malignancy. In addition, accumulating studies indicate that pyroptosis is involved in all stages of carcinogenesis. Thus, induction of pyroptosis could be considered an auspicious treatment compound for manipulating various cancers in the upcoming days (52).

The association involving pyroptosis with cancer is intricated, and the influences of pyroptosis on cancer are disparate based on depending on the tissues and genetic make-up of an individual. Pyroptosis presents two aspects of influences on multiple cancers. On the one side, the microenvironment created by pyroptosis modulates the process of tumor formation and progression including tumor growth, invasion, and metastasis. Chronic pyroptosis causes the liberation of the inflammatory cytokines IL-1, IL-18, LPH, and HMGB1, which could form an inflammatory microenvironment and actuate tumorigenesis. It has been reported in a review that NLRP3, IL-1β, and IL-18 motivate the development of tumors in lung cancer, melanoma, and breast cancer. Gao Tan also proved that HMGB1 could promote colorectal cancer tumorigenesis *via* the activation of the ERK 1/2 pathway (53). On the other side, pyroptosis induction suppresses the occurrence and development of cancers. Some researchists have elicited that several anti-cancer drugs can trigger pyroptosis to restrict malignancies. PPVI was reported by Jin-Feng Teng to markedly inhibit the growth of non-small cell lung cancer through the activation of caspase-1/GSDMD -mediated pyroptosis (54). In osteosarcoma, it has been demonstrated that Dioscin can inhibit tumor growth through GSDME-dependent pyroptosis (55). The main explication for the double-edged impacts of pyroptosis maybe is that inflammatory cytokines mediated by pyroptosis lead to chronic inflammation which propels the risk of oncogenesis (56) while acute pyroptosis activation results in cell mortality and restrains the progression of cancer (45). And beyond that, pyroptosis has advantages in overcoming chemotherapeutic drug resistance which is a deficiency in apoptosis. Pyroptosis inducers and chemotherapy regimens used together can improve the therapeutic efficacy. Qiao et al. (57) have substantiated that α-NETA triggered the GSDMD-mediated pyroptosis induced by caspase-4 in epithelial ovarian cancer. Several classical chemotherapy drugs including cisplatin, paclitaxel, 5-fluorouracil, and doxorubicin (DOX) was also reported to induce caspase-3/GSDME-modulated pyroptosis in various cancer cells (58–60). During the last several years, a considerable amount of small molecules currently were designed to execute pyroptosis in the cancer cell as therapeutic strategies. It thus appears that pyroptosis is quite significant in the development of cancer pathogenesis and cancer treatment. However, an extra exhaustive analysis of the processes and modulation of pyroptosis in cancer will favor the understanding of pyroptosis-related caner and develop more therapeutic targeting of pyroptosis.

## Pyroptosis and tumor immunity

Cancer immunotherapy achieves remarkable treatment effects in various types of cancer, but its efficacy for most tumors is still not well managed. The immune reaction of the tumor to immunotherapy is often reliant on the immunogenicity of cancer cells (61). Neoplastic cells with highly mutagenic can escape immunosurveillance, which generates tumors and resists anti-tumor immunity. Except for the immunostimulatory therapeutic regimens such as immune-checkpoint inhibitors (ICIs), adoptive cell transfer therapy, and dendritic cell-based vaccines, the attention of investigators is beginning to turn closer to the immunobiology of dying cancer cells. It is currently appreciated that pyroptosis-mediated therapy for anti-cancer which may either subdue or expand their immunogenic ability has already achieved notable success. The inflammatory reaction is closely connected to pyroptosis which has specific morphological features and drills into the cell membrane, causing the secretion of inflammatory factors, which ends up straight amplifying the systemic immune reaction.

As an inflammatory cell death, the immune system response brought on by pyroptosis has pro- and antitumorigenic effects in all periods of tumor occurrence. Pyroptosis activation and the secretion of cytokines related to pyroptosis either change the TME and accelerate cancer progression *via* employing immune evasion tactics to escape immune surveillance or stimulate the immunological system by igniting immune cells which generate immunological memory to achieve tumor regression and decrease the resistance to tumor immunotherapy (62). A previous study published by Pachathundikandi (63) demonstrated that Helicobacter pylori could induce pyroptosis *via* manipulating the generation of NLRP3 inflammasome and inflaming a mass of IL-1β or IL-18 release in human immune cells to reduce host immunity in gastric tumor development. Another study has confirmed that pyroptosis induced by NLRP3 and IL-1β secretion might adjust the TME towards an immune suppressive milieu, which facilitates cancer proliferation and invasion in mouse and human breast cancer (64). These study results suggested that inflammasomes trigged-pyroptosis can encourage immunosuppression or subvert immune response in TME, which provides the advantage to tumor cells eluding host cell immunologic reactions and provide protection against tumor progression. Instead, recent research has thrown new light on how immune cells undergo pyroptosis to expedite powerful immune reactions to exert their antineoplastic function. Sorafenib was demonstrated (65) that acts through direct immune modulation involving caspase-1-related MΦ pyroptosis and the following release of inflammasome-cytokine enhanced the cytotoxicity of NK cytotoxicity for the efficient tumor cell killing. Coincidentally, Yokoyama et al. (66) also revealed that the cotreatment of secretoglobin 3A2 and LPS significantly actuated pyroptosis of macrophages. This was urged by the caspase-11/NLRP3 inflammasome, which pressed on through immune regulation and decreased cancer cell proliferation *in vitro* and xenograft tumors in mice. Similarly, other researchers demonstrated that GSDMD levels were higher in activated CD8+ T cells and the lack of GSDMD could degrade their cytolytic capacity (67).

Apart from the analysis provided above, tumor-infiltrating immune cells were reported to active pyroptosis in cancer cells. Recently research discovered the GZM released from cytotoxic lymphocytes *via* the granule exocytosis pathway is crucial in pyroptosis. GZMA from the NK cell and CTLs were proven to induce pyroptosis in murine tumor cells *via* the cleavage of GSDMB, which not only enhances the antitumor immunity but also promotes tumor clearance (50). Similarly, Zhang et al. (49) have shown that GZMB interceded by killer cytotoxic lymphocytes shared the same cleavage site of GSDME with caspase-3 and triggered pyroptosis in many cancers, augmenting killer-cell immunity and enhancing the phagocytosis of tumor cells. Moreover, recent research showed that pyroptosis and ICIs work collaboratively to influence each other. Activation of pyroptosis in objective cells improves the sensitivity of the ICI-resistant cancers to checkpoint barricade and enhances antitumor activity (68). In mice bearing TNBC cell 4T1. Wang et al. (69) set up a bioorthogonal system applying GSDMA3 and anti-PD-1 mAb, which markedly sensitized to anti-PD-L1 cancer immunological therapy and decreased tumor growth. Last but not least, along with increased knowledge of the latent immunosuppressive mechanism of pyroptosis, several novel strategies for cancer therapy that drive cancer cells to pyroptosis and then increase the efficaciousness of cancer immunotherapy have achieved an unqualified success (70). Various new technologies and treatments can increase cancer risk cells to undergo pyroptosis, which can stimulate the immune system by promoting strong immune cell activity and accumulating and upregulating various immunological components to boost the effectiveness of immunotherapeutics. Veronica et al. (71) described a novel high-frequency irreversible electroporation technique to remove tumors, which inhibited 4T1 progression and stimulated a pro-inflammatory shift in TME related to pyroptosis. In turn, the level of tumor attenuation and metastatic lesions correlates with cellular immunity. Gao et al. (72) also substantiated the infusion of methotrexate-containing plasma-membrane microvesicles could induce GSDME-dependent pyroptosis of cholangiocarcinoma cells and the subsequent secretion of intracellular contents activate the production of proinflammatory cytokines from patient-derived macrophages, which ultimately stimulated a secondary wave of neutrophils to the tumor for the therapy of cholangiocarcinoma. Additionally, Fan et al. (73) introduced a novel strategy of combining decitabine which was able to reverse GSDME silencing with chemotherapy nanodrugs LipoDDP for causing cancer cells to activate the caspase-3 pathway and eventually stimulated the occurrence of pyroptosis. This combined chemotherapy-based pyroptosis promoted immunity cell activation and ulteriorly enhances the immunological effects of chemotherapy to annihilate the activities, metastasis, and recurrence of tumors.

These studies illustrated the effects of pyroptosis on tumor immunity are complicated. Pyroptosis-associated inflammasomes and cytokines are incorporated into tumorigenesis and the TME, which not only develop the tumor but also induce pyroptosis. The interesting thing is that pyroptosis can inhibit tumors and stimulate immune response which suppresses tumor escape in turn. In addition, the pyroptosis of immune cells also has a certain effect on tumors. All in all, the two-way connection between tumor immunocompetence and pyroptosis is going to be a promising investigation of hot issues for tumor immunotherapy.

## Searching strategy

To identify the eligible studies about the current molecular mechanisms of pyroptosis in breast cancer, we have conducted a systematic search in four common databases [PubMed, Cochrane Library, EMBASE (OVID), and PsychINFO] prior to June 1, 2022. The searching strategy was: [(“Breast Neoplasms”[Mesh]] OR (((((((((((((((((((((((((((((((((((((Breast Neoplasm) OR (Neoplasm, Breast)) OR (Breast Tumors)) OR (Breast Tumor)) OR (Tumor, Breast)) OR (Tumors, Breast)) OR (Neoplasms, Breast)) OR (Breast Cancer)) OR (Cancer, Breast)) OR (Mammary Cancer)) OR (Cancer, Mammary)) OR (Cancers, Mammary)) OR (Mammary Cancers)) OR (Malignant Neoplasm of Breast)) OR (Breast Malignant Neoplasm)) OR (Breast Malignant Neoplasms)) OR (Malignant Tumor of Breast)) OR (Breast Malignant Tumor)) OR (Breast Malignant Tumors)) OR (Cancer of Breast)) OR (Cancer of the Breast)) OR (Mammary Carcinoma, Human)) OR (Carcinoma, Human Mammary)) OR (Carcinomas, Human Mammary)) OR (Human Mammary Carcinomas)) OR (Mammary Carcinomas, Human)) OR (Human Mammary Carcinoma)) OR (Mammary Neoplasms, Human)) OR (Human Mammary Neoplasm)) OR (Human Mammary Neoplasms)) OR (Neoplasm, Human Mammary)) OR (Neoplasms, Human Mammary)) OR (Mammary Neoplasm, Human)) OR (Breast Carcinoma)) OR (Breast Carcinomas)) OR (Carcinoma, Breast)) OR (Carcinomas, Breast))) AND ((“Pyroptosis”[Mesh]) OR ((((((((((((Pyroptoses) OR (Pyroptotic Cell Death)) OR (Cell Death, Pyroptotic)) OR (Death, Pyroptotic Cell)) OR (Deaths, Pyroptotic Cell)) OR (Pyroptotic Cell Deaths)) OR (Caspase-1 Dependent Cell Death)) OR (Caspase 1 Dependent Cell Death)) OR (Inflammatory Apoptosis)) OR (Apoptoses, Inflammatory)) OR (Apoptosis, Inflammatory)) OR (Inflammatory Apoptoses))). To find additional relevant research, we also examined the reference lists in the related articles manually.

**Figure 2:**
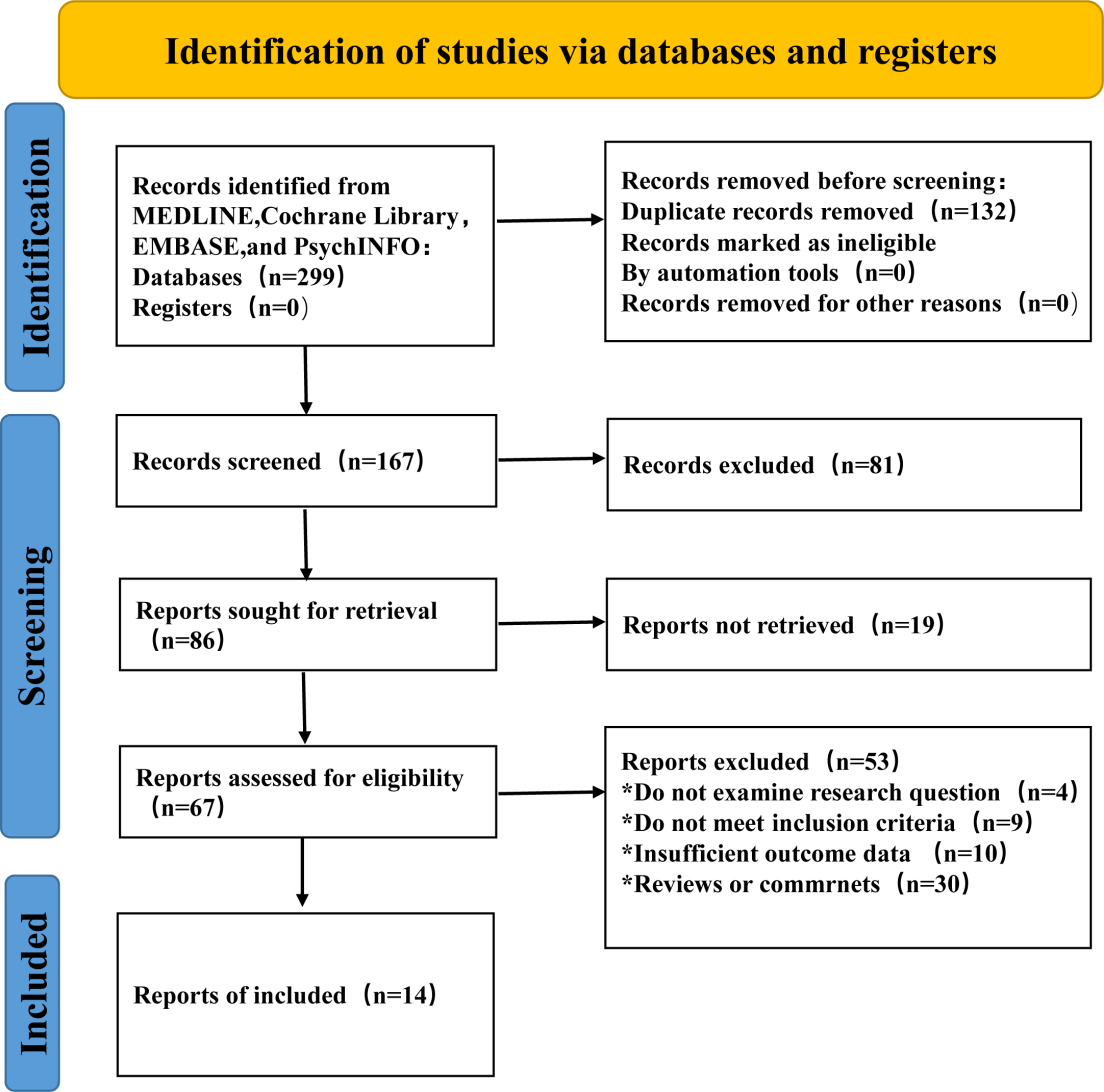
Flow chart of study selection to identify the relevant studies reported on the association between pyroptosis and breast cancer.

**Figure 2** showed the search flowchart for identifying the eligible studies reporting the relationship between breast cancer and pyroptosis. In the initial database search, 299 publications were detected, of which 67 came from PubMed, 147 from EMBASE, 75 from the Cochrane Library, and 10 from the PsychINFO database. After excluding duplicates and those studies with reasons, 14 eligible studies (42, 45, 77, 78, 83, 86, 91, 93, 97, 103, 106, 107, 108, 109) were finally included. **Table 1** summarizes the biomolecular mechanisms of pyroptosis in breast cancer mentioned in the 14 included studies.

**Table 1:** Mechanisms of pyroptosis in breast cancer.

Reference	Model	Biomolecular mechanism
(42)	MDA-MB-231 cell 4T1 cell BT-549 cell (mouse model with MDA-MB-231 cells)	SSP promotes inflammasome caspase-4/9 to cleave GSDME, which causes pyroptosis and membrane permeabilization.
(45)	MDA-MB-231 cell 4T1 cell (mouse model with MDA-MB-231 cells and 4T1 cells)	PD-L1 switches TNFα-derived-apoptosis to caspase-8-mediated pyroptosis under hypoxia.
(74)	MDA-MB-231 cell 4T1 cell	Docosahexaenoic acid leads to NF-κB nuclear translocation and induces caspase-1/GSDMD dependent pyroptosis.
(75)	MCF-7 cell BT-549 cell	Nobiletin promotes the caspase-1/GSDMD pyroptosis *via* the regulation of the miR-200b/JAZF1 axis.
(76)	MDA-MB-231 cell (mouse model with MDA-MB-231 cells)	Cisplatin activates pyroptosis by activating MEG3/NLRP3/caspase-1/GSDMD axis.
(77)	4T1 cell (mouse models with 4T1 cells)	By blocking the JAK2/STAT3 signaling pathway, polydatin increases the pyroptosis in a caspase-1/GSDMD manner.
(78)	MCF-7 cell MDA-MB-231 cell (mouse model with MDA-MB-231 cells)	Dihydroartemisinin causes pyroptosis *via* the activation of the AIM2/caspase-3/GSDME axis.
(79)	Hs578T cell BT-549 cell 4T1 cell (mouse model with 4T1cells)	Transfection of poly I: C promotes caspase-3/GSDME dependent pyroptosis by the suppression of TGF-β signaling depending on MDA5- and RIG-I.
(80)	MDA-MB-231 cell	Acute cadmium exposure can cause Bax to become activated, which in turn triggers caspase-3-mediated GSDME-mediated pyroptosis.
(81)	MDA-MB-231 cell T47D cell	Doxorubicin triggers caspase-3/GSDME-related pyroptosis *via* the ROS/JNK axis.
(82)	MCF-7 cell MDA-MB-231 cell (mouse model MDA-MB-231 cells)	Triclabendazole causes caspase-3 to become activated and cleave GSDME, which induces pyroptosis.
(83)	MDA-MB-231 cell 4T1 cell Hs578T cell EO771 cell	Tetraarsenic hexoxide activates caspase-3/GSDME by enhancing the generation of mitochondrial ROS.
(84)	MDA-MB-231 cell BT-549 cell	Overexpression of mitochondrial protein UCP1 activates caspase-3/GSDME-dependent pyroptosis.
(85)	MDA-MB-231 cell 4T1 cell BT549 cell YCCB1 cell	DRD2, a tumor suppressor, encourages M1 macrophages and limits NF-B signaling to cause pyroptosis.

(Mouse cells: 4T1 cell, EO771 cell; Human cells: MDA-MB-231 cel1, MCF-7 cells, BT549 cell, YCCB1cell, Hs578T cell, T47 cell).

## Molecular mechanism of pyroptosis in breast cancer

The central mediators of pyroptosis are proteins from the Gasdermin family (86).

**Figure 3:**
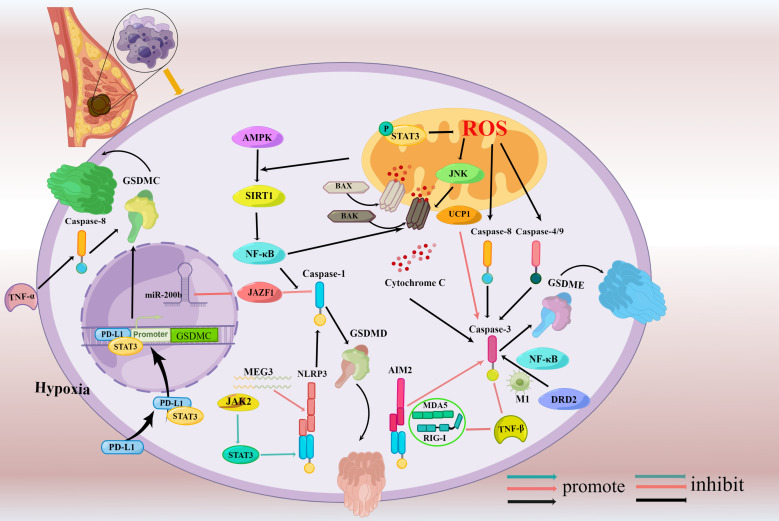
Signaling pathways regulating pyroptosis in breast cancer. (Mechanisms involving only mouse cells were drawn in blue indicator lines while human cells were drawn in red indicator lines, and mechanisms involving both mouse cells and human cells were drawn in black indicator lines.) Caspase-3/GSDME-dependent pyroptosis is induced through activating molecules including Bax, AIM2, caspase-4, caspase-9, DRD2, and UCP1 and AMPK/SIRT1/NF-κB/Bax axis and STAT3/ROS/JNK axis involved in the process. Besides it, the suppression of TGF-β signaling *via* MDA5- and RIG-I- also trigger caspase-3/GSDME dependent pyroptosis. MEG3/NLRP3, miR-200b/JAZF1, JAK2/STAT3 signaling pathway, and NF-κB participate in the caspase-1/GSDMD-pyroptosis.PD-L1 under hypoxia switches TNFα-derived-apoptosis to noncanonical pyroptosis triggered by caspase-8 and is dependent on GSDMC.

The majority of previous research about pyroptosis concentrated on caspase-1/4/5/11 and GSDMD, and this kind of cell death was frequently referred to as inflammatory cell death. However, scientists focused their emphasis on another gasdermin family which directly ruins the cell membranes and serve as executor in pyroptosis with the intensive investigation recently (86). Six categories of gasdermins have been identified which have different roles in breast cancer respectively (**Table 2**) and the specific molecular mechanism of pyroptosis in breast cancer will be briefly introduced below through the six members of the gasdermins. Among these, GSDMD and GSDME have been thoroughly examined in pyroptosis, and the two executioners will be investigated in depth below (**Figure 3**).

**Table 2:** The introduction and features of GSDMs.

HumanGSDMs	Mouse homolog	Pore-forming activity	Activating proteolytic cleavage	Relationship withBreast cancer	References
GSDMA	GSDMA1-3	Yes	Uncertain	Anti-oncogene	(69)
GSDMB	Absent	Yes	Caspase-1/-3/-6/-7/ Granzyme A	Oncogene	(87–89)
GSDMC	GSDMC1-4	Yes	Caspase-8	Oncogene	(45)
GSDMD	GSDMD	Yes	Caspase-1/-4/-5/-11/-8	Uncertain	(90)
GSDME/DFNA5	GSDME/Dfna5	Yes	Caspase-3/Granzyme B	Anti-oncogene/Oncogene	(91, 92)
DFNB59	DFNB59	Uncertain	Uncertain	Uncertain	None

The following is explored in depth with these two executioners, GSDMD and GSDME, two of these molecules that have been thoroughly examined in pyroptosis.

## Potential strategies targeting GSDMD-mediated pyroptosis

The oldest known gasdermin related to pyroptosis is GSDMD. Caspase-1/4/5/11 in the canonical pyroptosis pathway and caspase-8 in the non-canonical pyroptosis pathway may both cleave GSDMD. According to a spearman correlation analysis of breast cancer and adjacent noncancerous growths carried out by Wu et al. (90), there was positive relevancy among the expression of GSDMD, caspase-1, and IL-1β, which is compliant with the GSDMD regulated pyroptosis development law. Meanwhile, breast cancer pathological grade and TNM staging were adversely connected with the levels of pyroptosis-associated proteins expression, which concludes that the occurrence, growth, metastasis, and prognosis of breast cancer are significantly influenced by GSDMD.

## NF-κB/caspase-1/GSDMD

Nuclear factor-kappa B (NF-κB), a factor controlling DNA transcription, is well recognized for its control over cell division and involvement in inflammatory immune responses (93). Pizato et al. (74) demonstrated that Dihydroartemisinin (DHA) triggered diverse pyroptosis biomarkers in TNBC cells MDA-MB-231 from humans and murine TNBC cells 4T1, which induced pyroptosis eventually. The results of the study showed that DHA first causes NF-κB nuclear translocation and an increase in ASC expression. Then, after being exposed to DHA, breast cancer cells secreted more IL-1β and moved HMGB1 from the nucleus to the cytoplasm *via* activating caspase-1 and GSDMD. Moreover, Wang et al. (75) confirmed that nobiletin can inhibit breast tumor growth by inducing pyroptosis in miR-200b/JAZF1/NF-κB manner.

## MEG3/NLRP3/caspase-1/GSDMD

As emerging regulators of cell pyroptosis, long noncoding RNAs (lncRNAs) have drawn widespread interest recently (94, 95). As a type of lncRNA, maternally expressed gene 3 (MEG3) has been studied extensively in oncogenesis and actions. And a recent study found that MEG3 controlled the MEG3/miR-223/NLRP3 axis to prevent pyroptosis (96). Another research proved that MEG3 knockdown mitigated lung damage induced by hyperoxia by blocking pyroptosis (97). All those evidences revealed that MEG3 participated in the process of pyroptosis. Honglin Yan (76) also showed that Cisplatin (DDP) had the effect of an anti-tumor on TNBC, which is brought on by up-regulating MEG3 to cause pyroptosis in NLRP3/caspase-1/GSDMD manner. Similarly, the activating impact of DDP on pyroptosis was also eliminated by MEG3 knockdown, which indicates that MEG3 is crucial for pyroptosis in breast cancer.

## JAK2/STAT3/NLRP3/caspase-1/GSDMD

Previous studies have shown that JAK/STAT axis is pivotal in cell metabolism and mammary cancer cell proliferation (98). And it has been noted that the stimulation of JAK2/STAT3 causes the breast stemness gene expression and could be a novel prognostic indicator for metastasis of breast cells (99). Lately, Liu et al. (77) hypothesized that polydatin has an anti-cancer effect on TNBC mice fed a fat-rich diet *via* inducing pyroptosis in JAK2/STAT3 manner. In the experimental models, polydatin downregulated the phosphorylation of STAT3 and JAK2 and enhanced the expression of NLRP3, caspase-1, IL-1β, and IL-18 to take part in the activation of pyroptosis.

## Potential strategies targeting GSDME mediated pyroptosis

GSDME, also called DFNA5 was originally discovered as a gene for hearing loss because of the association between mutation and a specific autosomal dominant non-syndromic in deafness 1988 (100). In ER-negative breast cancer, GSDME is overexpressed and may be involved in the carcinogenesis independent of hormonal response (91). Compared to matching normal breast tissue, Kim et al. (92) found that the GSDME promoter frequently methylates in primary breast cancer tissue samples, and this methylation generally reduced the gene expression of GSDME. The study also showed that only cell lines with estrogen receptor-positive were found to have this methylation. Additionally, in breast cancer patients, the methylation status of GSDME was linked to lymph node metastases. Thus, the right chemotherapy agents for the therapeutics of breast cancer should be chosen based on levels of GSDME expression to enhance the sensitivity to chemotherapy medications and reduce drug resistance. According to recent reports, the cleavage of GSDME is facilitated by chemotherapy medicines *via* activating caspase-3 which is a key apoptosis executor, eventually switching apoptosis to pyroptosis as secondary necrosis (21, 44). Even more to the point, these cells participate in pyroptosis only in presence of GSDME. Thus, chemotherapy drugs can induce GSDME-expressing breast cancer cells to undergo caspase-3-triggered pyroptosis (44).

## AIM2 or MDA5 or RIG-I/caspase-3/GSDME

AIM2, a part of the inflammasome involved in pyroptosis, can stimulate caspase-1 and caspase-8 activity at the same time and cause the caspase-3 to be split (101). A recent study reported by Yaqiong Li (78) indicated that breast cancer cells undergo pyroptosis *via* the AIM2/caspase-3/GSDME pathway active by DHA. In terms of the mechanism, DHA stimulated the production of AIM2, and AIM2 upregulated the expression of GSDME by triggering caspase-3. In addition, among PRRs which consist of inflammasomes, it is hypothesized that the tumor growth was inhibited by activating retinoic acid-inducible gene-I (RIG-I)-like receptors (RLRs) like melanoma differentiation-associated gene 5 (MDA5) and RIG-I (102). Transfection of poly I: C, a commonly utilized artificial double-stranded RNA (dsRNA) analog, has been probed to activate RLRs and evaluated in clinical trials. In a recent study, Yusuke Tamura (79) showed that transfection of poly I: C inhibited transforming growth factor-β (TGF-β) signaling *via* MDA5 and RIG-I and promoted caspase-3/GSDME dependent pyroptosis in TNBC. Moreover, the pyroptosis could be attenuated by forcing constitutively active Smad3 expression, which is phosphorylated by downstream components of TGF-β signaling. And additional RLR ligands for the treatment of cancer may exhibit semblable repressive functions on TGF-β signaling and enhance pyroptosis in the light of the findings.

## BAK or BAX/caspase-3/GSDME

Some antitumor therapeutic regimens caused the cell death of cancer in the mitochondrial manner regulated by BCL2 family proteins. BAK and BAX are BCL2 family members that can generate holes on the outer membrane of the mitochondrial after activation, leading to the secretion of constituents such as cytochrome C inside the mitochondrial membrane and activating the caspase cascade (44, 103, 104). An experiment conducted by Lei Hu (105) proved that breast cancer cells were stimulated to undergo pyroptosis *via* BAK or BAX/caspase-3/GSDME axis with the treatment of TNFα+CHX and navitoclax. Furthermore, the C-terminal of GSDME was discovered to be palmitoylated by several ZDHHC proteins to stimulate its dissociation from N-terminal, which escalated chemotherapy drug-induced pyroptosis. The study supported not only the notion that the activation of BAK and BAX regulate the cleavage of GSDME but also that either solitary BAK or BAX activation might trigger the caspase-activating chain and following pyroptosis processes. Likewise, Tang et al. (80) demonstrated that Cadmium (Cd) exposure can give rise to GSDME-dependent pyroptosis in TNBC, and the activation of the Bax/caspase-3 axis is crucial to this activity. Furthermore, several drugs can activate BAX to participate in the pyroptosis of breast cancer and exert its anticancer effects. Metformin, a widely prescribed medicine to treat diabetes, reportedly promoted AMP-activated protein kinase (AMPK) activation, which is vital in various cancer cachexia and its downstream signaling pathway (106). SIRT1, an NAD+-dependent deacetylase, modulates NF-B to serve a variety of tasks in an immune reaction and inflammatory processes (107). A previous study revealed that metformin could derive caspase3/GSDME-mediated pyroptosis and significantly increase LDH levels in breast cancer by enhancing AMPK/SIRT1/NF-κB/Bax signaling (108). The underlying mechanisms were that metformin activated AMPK and induced mitochondrial dysfunction. On one hand, activated AMPK also caused SIRT-1 and NF-B p65 to be raised, encouraging the activation of Bax and the liberation of cytochrome C, which then initiated caspase-3 and the formation of GSDME-N and finally lead to pyroptosis. Metformin can also boost the manufacturing of reactive oxygen species (ROS) in the mitochondria, which will favorably upregulate the expression of Bax. Additionally, metformin therapy causes cancer cells to undergo pyroptosis mediated by caspase3/GSDME when mitochondrial dysfunction triggers the AMPK/SIRT1 pathway.

## ROS/caspase-3/GSDME

ROS which is the active form of oxygen has been shown to monitor apoptosis and autophagy in cancer cells (109) and is associated closely with the caspase-GSDME pathway (81, 110). The MAPK signaling pathway has been included as one of the pathways controlled by ROS (111). JNK, a member of the MAPK family of stress-activated protein kinases, is essential for several cellular processes (112). Yan et al. (82) revealed that triclabendazole could induce pyroptosis involving caspase-3, GSDME, and the mitochondrial apoptotic mediated by ROS/JNK/Bax axis in breast cancer cells. Additionally, Zhang et al. (81) suggested that caspase-3-regulated GSDME caused pyroptosis *via* the ROS/JNK or ROS/caspase-8 signaling pathway with the treatment of DOX in breast cancer cells lately. The accumulation of ROS triggered by DOX was able to promote the phosphorylation of JNK or the cleavage of caspase-8. Both p-JNK and caspase-8 could further activate caspase-3 through a cascade reaction. Haein An (83) also showed that tetraarsenic hexoxide inhibited the development and metastasis of TNBC *via* activating the mitochondrial ROS-mediated caspase-3/GSDME axis and inducing pyroptosis by preventing the mitochondrial STAT3 activation. Besides it, Zhang et al. (42) uncovered that SSP upregulated ROS generation and elevated caspase-4 and -9, subsequently cleaved GSDME and induced pyroptosis in breast cancer. While GSH, a suppressant of ROS, notably attenuated the ROS triggered by SSP and rescued pyroptosis.

## Other pathways

Jing Xia (84) demonstrated that a high level of mitochondrial protein UCP1 caused mitochondrial damage and malfunction and inhibited the proliferation and malignant characteristics of TNBC depending on caspase-3/GSDME mediated pyroptosis. Furthermore, a study reported by Yiqing Tan (85) novelly manifested the role of DRD2 in suppressing breast cancer tumorigenesis and further revealed that DRD2 educated M1 macrophages and restricted the NF-κB pathway, triggering pyroptosis in breast cancer.

## Potential strategies targeting GSDMB mediated pyroptosis

The only gasdermin member absent from the rodent genome is GSDMB (also called PRO2521, GSDML in the past) (113). Four GSDMB splice variants have been identified and each one could have a unique function in cancer. Of particular attention here is that GSDMB prefers to attach to membranes differently than other gasdermins. The phosphoinositide of the cell membrane can be contacted by both the whole length of GSDMB and the N-terminal domain (114). Marta Hergueta-Redondo (87) uncovered the initial functional significance of GSDMB in breast cancer and the overexpression of GSDMB are closely linked to increased metastasis and reduced survival in breast cancer patients. What’s more, in HER2− breast cancer patients, GSDMB expression correlates with elevated metastasis and bad outcomes of patients (88). Those all manifest that GSDMB may be a novel evaluation and prognosticate marker for breast cancer. Regarding the activation of GSDMB, several studies suggested that apoptotic rather than inflammatory caspase-3/6/7 can cleave GSDMB while other investigations reported that caspase-1 is responsible for the GSDMB cleavage (89, 114). Besides it, it also has been reported that GZMA can cleave GSDMB in murine cancer cells recently (50).

## Potential strategies targeting GSDMC mediated pyroptosis

In individuals with breast cancer, a high level of GSDMC was connected to a bad prognosis. Junwei Hou (45) showed that PD-L1 under hypoxia switched TNFα-derived-apoptosis to pyroptosis mediated by noncanonical caspase-8 in MDA-MB-231 and 4T1 cells, eventually contributes to tumor necrosis. The correlation research indicated hypoxic stress can initiate tumor necrosis because of accelerated tumor development and insufficient blood flow and TNF-α has long been recognized to engender cancer necrosis (89). In breast cancer, hypoxic stress-activated p-Y705- STAT3 is physically linked to PD-L1 and facilitated its strong translocation of nuclear through the importin α/β signaling (45). To transcriptionally stimulate GSDMC expression, PD-L1 formed a complex with p-Y705-STAT3 and bound to the STAT3-binding site of the GSDMC promoter. Then TNFα-activated caspase-8 preferentially cleaved GSDMC, lysing GSDMC to produce GSDMC-N which eventually induces pyroptosis. The study (45) also implied that treatments consisting of one of the four antibiotics (epirubicin, daunorubicin, actinomycin-D, and doxorubicin), may result in significant inflammatory that may have an impact on the survival and anticancer immunotherapy of GSDMC+ cancer patients.

## Potential strategies targeting other GSDMs mediated pyroptosis

There is only one subtype of GSDMA in the human while the murine gene expresses three paralogs designated as GSDMA1, GSDMA2, and GSDMA3. In 2018, the crystal structure of the mouse GSDMA3 pore was settled (115), which offered vital insight into the cytomembrane pore formed by gasdermins. Wang et al. (69) fabricated a bioorthogonal chemical system to aggregate and release the active GSDMA3 to breast cancer 4T1 cells, which not only provoked pyroptosis but also enlarged immune response. Nonetheless, there is currently a paucity of research between GSDMA and breast cancer. Further research is needed to determine whether GSDMA can cause pyroptosis through any undiscovered mechanisms. As mentioned, most gasdermins possess a uniform architecture except DNFB59 whose C-terminal domain has been cut off. Its mutation is concerned with auditory neuropathy through expression in inner ear hair cells. While there isn’t yet evidence linking DNFB59 to breast cancer and it also has not been proven to cause pore generation.

## Relationship between pyroptosis and breast cancer immune regulation

Cancer cell death triggered by certain initiating stimuli can be immunogenic and is commonly known as immunogenic cell death (ICD). Pyroptosis can elicit a robust inflammatory response and is considered a newly characterized form of ICD in some cases (49). More and more research has suggested that controlled activation of pyroptosis can induce anti-tumor immunity (50, 69). In the following sections, the relationship between pyroptosis and breast cancer immunity is described according to the inflammasomes, gasdermins, and inflammatory cytokines involved in the process of pyroptosis.

## The modulatory effects of inflammasomes from pyroptosis on immunity

**Figure 4:**
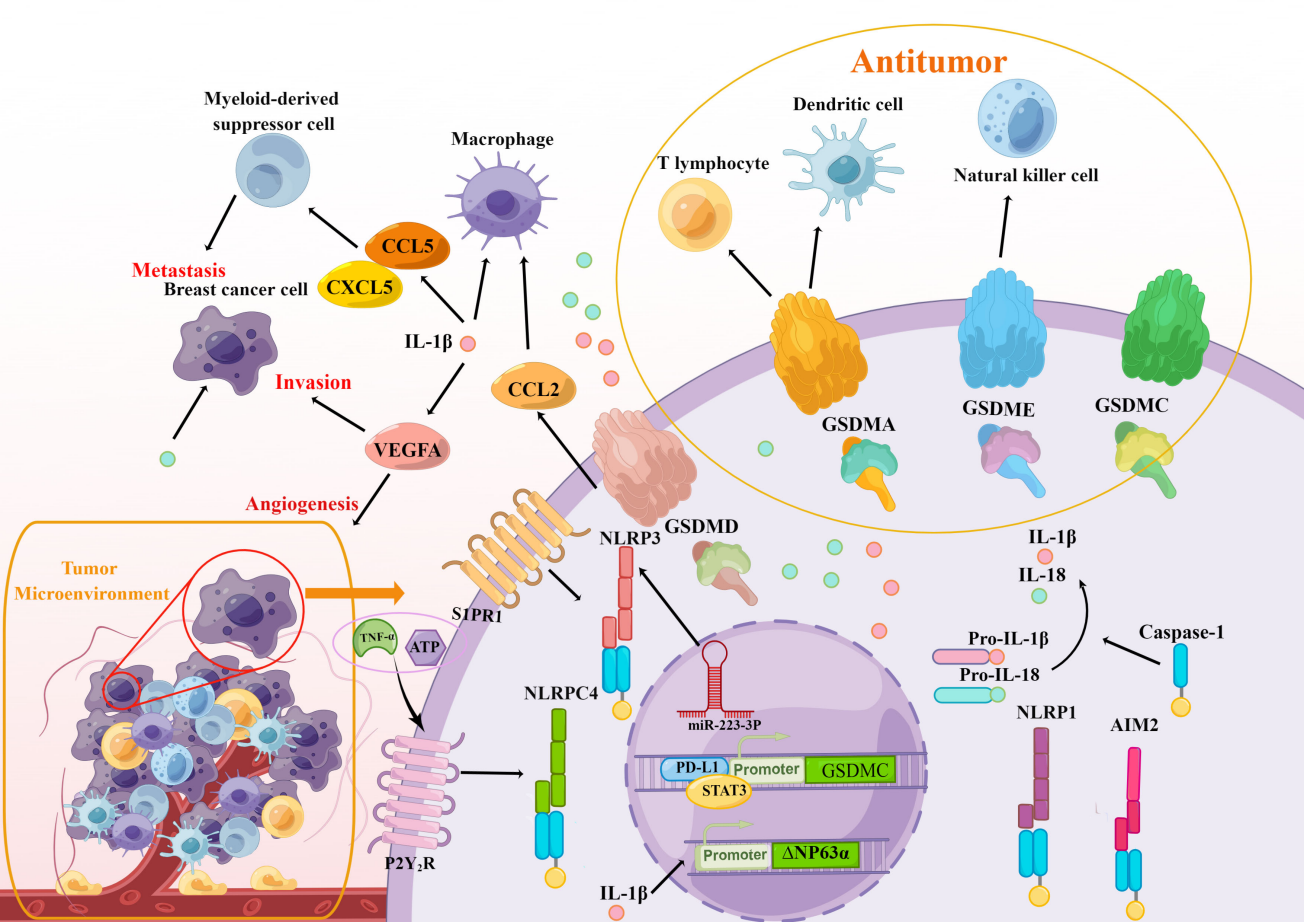
The modulation of breast cancer immunity is associated with pyroptosis. The crucial elements in the pyroptosis pathways, including inflammasomes (NLRPC4, NLRP3, NLRP1, AIM2), gasdermins (GSDMA/C/D/E), and inflammatory cytokines IL-1β and IL-18 take part in the initiation and progression of immune response in the TAM of breast cancer and affect the immune cells including macrophage, myeloid-derived suppressor cell, T lymphocyte, dendritic cell and natural killer cell, which promote the metastasis, invasion, and angiogenesis of breast cell. At the same time, active gasdermins delivered directly to breast cancer cells will greatly enhance immunotherapy.

Tschopp and colleagues (19) identified a caspase-activating complex as an inflammasome in 2002, which was the first time the idea of inflammasomes was raised. Inflammasomes are multiprotein signaling platforms that respond to pathogenic microorganisms and endogenous danger signals through innate immunity, leading to inflammation and pyroptosis, which coordinate antimicrobial host defenses (116). Inflammasomes are composed of sensor and adaptor proteins. As presented above, inflammasome sensors PRRs, which are innate immune receptors and associated with tissue damage and pathogen infection, are grouped based on their structural features into NLRs, AIM2, and Pyrin (30). The NLR family includes several subfamilies divided into NLRP or NLRC based on N-terminal effector domains which contain PYD or CARD (117). In particular, *via* controlling innate and adaptive immunity as well as tumor growth, NLRP1, NLRP3, NLRC4, and AIM2 have an effect on how breast cancer develops. (**Figure 4**).

## NLRP3

It has been proven that the level of NLRP3 was aberrantly high in the microenvironment and the NLRP3 activation enhanced tumorigenesis and metastasis as well as the invasion of myeloid cells like tumor-associated macrophages (TAMs) and myeloid-derived suppressor cells (MDSCs) in breast cancer orthotopic models (118). In addition, the researchers uncovered that the NLRP3 inflammasome promoted the infiltration and metastasis of lymph nodes in patients with HER2+ breast cancer through the lymphatics downstream of S1PR1 signaling in macrophages (119). From that, one hypothesis is that the inhibition of the NLRP3 may impede breast cancer tumorigenesis. Hu et al. (120) investigated the response of polymeric nanocarriers *in vivo* immune and the results showed that administration of PEI 25 kD could induce high oxidative stress and NLRP3-inflammasome activation, which greatly promoted breast cancer metastasis in liver and lung tissues. Likewise, Zhang et al. (121) hypothesized that miR-223-3p may make a suppressive effect on breast cancer proliferation and immunosuppression *in vitro* and *in vivo* by inactivating the NLRP3 inflammasome.

## NLRP1

Yuxian Wei (122) discovered that primary breast cancer tissue had higher levels of NLRP1 expression compared to neighboring non-cancerous tissue and there was a link between NLRP1 expression and clinical indexes such as Ki-67 levels, TNM stage, and lymph node metastasis. Moreover, the high level of NLRP1 expression in MCF-7 cell facilitated the proliferation, migration, invasion, and tumorigenicity of mice by inducing epithelial-mesenchymal transition (EMT). Besides it, Jiao et al. (123) speculated that the secreted factors of hUCMSCs were able to induce pyroptosis in the MCF-7 cell *via* upregulating NLRP1 and caspase-4 according to the RNA sequencing studies. And classified 14 significant pathways identified by KEGG analysis and found that they are mainly related to the immune system. Next, Jiao et al. (124) further confirmed that the pyroptosis caused by hUCMSC-CM in MCF-7 cells is induced by the NLRP1 inflammasome complex through relevant experiments. However, the mechanisms of how NLRP1 exerts its tumor suppression effect in the immune system need further research.

## NLRC4

The differential expression of NLRC4 has investigated the potential role of NLRC4 in different kinds of tumor types. More and more studies have attested that higher NLRC4 expression in breast cancer and glioma (125). Hana Jin and Hye Jung Kim (126) determined that NLRC4 inflammasome regulated by tumor necrosis factor-α (TNF-α) or ATP dependent on P2Y2R is engaged in breast cancer cells invasion and angiogenesis Moreover, breast cancer patients who are obese tend to have more myeloid cells that infiltrate tumors, which activated NLRC4 and mature IL-1β. Obesity-associated NLRC4 inflammasome activation could also mediate the adipocyte-mediated vascular endothelial growth factor A (VEGFA) expression and angiogenesis, which speed up the course of invasion in breast cancer (127).

## AIM2

A study reported by I-Fen Chen (128) demonstrated that the expression of AIM2 restrained breast cancer tumorigenicity and proliferation *in vivo* and *vitro*. Furthermore, combined with innate immune agonists, high intensity focused ultrasound (HIFU) in conjunction with innate immune stimulators could upregulate multiple innate immune receptors including AIM2 in mice with multi-focal breast cancer, which enhanced response to innate immune agonists (129). Su et al. (130) also reported an unexpected finding that AIM2 is recruited to the phagosomes and activated following antibody-dependent cellular phagocytosis (ADCP), which subsequently caused immunosuppression in HER2+ breast cancer. Recently, Yaqiong Li (101) found that DHA inhibited tumorigenesis by inducing AIM2/caspase-3/GSDME regulated pyroptosis in breast cancer cells. Those all indicated a potent antitumor activity of AIM2 and its association with immunity in breast cancer.

These researches indicate that the high expression and activation of inflammasomes NLRP3, NLRP1, and NLRC4 may promote breast cancer progression including growth, metastasis, and invasion. Given the inhibition of NLRP3 inflammasome in blocking breast cancer progression, various agents inhibiting inflammasomes can be utilized for therapeutic strategies. Inflammasomes are closely related to immune response while the studies about the relationship between immunotherapy of breast cancer and inflammasomes are scarce, which is a gap that needs to be filled.

## The effect of the gasdermin family on the modulation of immunity

As mentioned above, a variety of researches indicate that targeted delivery of bioactive gasdermin molecules to the tumor can induce impressive antitumor immunity. Additionally, the GSDME and GSDMB are cleaved directly by granzyme in cancer cells also, in turn, enhancing anticancer immunity (49, 50, 69) (**Figure 4**). Wang et al. (69) establish a bioorthogonal chemical technique to release bioactive GSDMA into tumor cells and discovered that less than 15% of cancer cells’ pyroptosis was adequate to eradicate the entire 4T1 mammary tumor graft. While in immunodeficient mice, this clearance of the tumor vanished, which revealed that GSDMA could be essential for augmenting antitumor immune function. As shown in research, PD-L1 (45) enhanced the expression of the GSDMC following hypoxia, and caspase-8 then specifically cleaved the GSDMC, which caused the TNFα derived from macrophages to trigger breast cancer pyroptosis *in vivo* and inhibit antitumor immunity in addition to facilitating tumor development. In addition, the antitumor immunity of GSDMC+ cancer patients were also related to the strength of the treatment with inflammation caused by antibiotics. GSDMD was also proved to mediate pyroptosis and effectively stimulate tumor immunogenicity, promoting the maturation of dendritic cells (DC) cells and fully activating T cells reliant adaptive immune responses in TNBC, ultimately eradicating distant cancers while killing the original tumor (131). GSDME reportedly exerts positive effects on immunity in cancer. Zhang et al. (49) observed that GSDME was able to increase the amount and capabilities of NK cells and CD8+ lymphocytes to engulf cancer cells whereas, in the TME of the GSDME -/- murine TNBC cell, the infiltration of CD8+ lymphocytes and NK cells reduced. Zhao et al. (132) constructed a biomimetic nanoparticle-containing indocyanine green and decitabine, which up-regulated GSDME expression synergistically by inhibiting the methylation of DNA. Then the BNP facilitated caspase-3 cleavage to GSDME and caused pyroptosis of breast cancer cells and followed by a robust systemic antitumor immunity for the suppression of growth and distant tumor metastasis. In summary, gasdermins are essential in the TME of breast cancer and more novel strategies should be explored to deliver active gasdermins directly to breast cancer cells, which will greatly enhance immunotherapy in breast cancer.

## Immune system modulation by inflammatory cytokines produced by pyroptosis

Pyroptosis is distinguished by the secretion of inflammatory cytokines IL-1 and IL-18 from GSDMD-forming holes, as well as the inflammatory reactions brought on by inflammasomes. after pyroptotic cell rupturing (36). Multitudinous studies showed that IL-1β and IL-18 also serve an essential function for the immune system (133). The function of IL-1β and IL-18 in breast cancer are multiple as described above. On one hand, the expression of inflammatory cytokines transports immune cells into the TME, which stimulates the development of breast cancer. In addition, inflammatory cytokines can stimulate immunity cells and immunological cytokines, suppressing the tumorigenesis, growth, and metastasis of breast cancer cells (**Figure 4**).

Most malignancies, including breast cancer, have elevated levels of IL-1β which is bounteous in the TME, in which it can avail tumor proliferation, but also antitumor activities (134). Besides this, IL-1β also make an effect on immunity such as accelerating adaptive T cell-mediated immunity and promoting CD4+ and CD8+ T cells maturation (135). The translocation of MDSCs and TAMs into the TME was triggered by NLRP3-mediated pyroptosis and the liberation of IL-1 at primary and metastatic locations, which induced the growth and metastasis of human breast cancer (118). Another study also demonstrated that NLRP3 pyroptosis caused IL-1β maturation and the resulting CCL5, CXCL12, CCL2, and CXCL5 expression, which enhanced metastasis of breast cancer by recruiting MDSC and M2 macrophages (136). On the contrary, Guo et al. (137) discovered that innate immune cells that invade remote metastasis-initiating cancer cells (MIC) microenvironments could express IL-1β and evoke a systemic inflammatory response in certain primary tumors. In individuals with lymph node-positive breast cancer, improved prognosis, and distant metastasis-free outcomes are strongly correlated with high primary tumor IL-1 expression. This shows that breast cancer is affected differently depending on whether IL-1 is expressed by immune cells or tumor cells. In addition, the environment with inflammatory infiltrates is indispensable for the generation of drug resistance in cancer cells. It was disclosed that IL-1β could enhance the tumor protein 63 (TP63) isoform ΔNP63α, a chemoresistance-associated gene, adding to the cisplatin acquired resistance in breast cancer cells (138). While Irena Kaplanov (139) also demonstrated that blocking IL-1β facilitates immunosuppression in the TME of first-generation orthotropic mammary cancers. Apart from that, IL-1β inhibition acted in synergy with anti–PD-1 could lead to the restoration of the T cell-mediated tumor immunity for optimal tumor killing.

IL-18 was initially identified for its propensity to cause anti-CD3-stimulated T cells to create an IFN-induing factor.to produce IFN-γ-induing factor from anti-CD3-stimulated T cells (140). It is broadly considered that IL-18 serves as a key executor in launching anticancer immune functions such as modulating immune system components through attracting or differentiating NK cells, T cells, monocytes, and so on (141, 142). By triggering immune cells and immune cytokines, human mesenchymal stem cells from the umbilical cord (hUMSCs)-expressing IL-18 have been shown to suppress the growth, invasion, and metastasis of breast cancer cells *in vitro*, lowering the proliferation index of the marker Ki-67, and halting tumor progression (143). Conversely, IL-18 also comes to terms with tumor immune responses to support cancer evasion. IL-18 derived from breast cancer promoted PD-1 expression in NK cells and increased their immunosuppressive fraction, which is connected to bad outcomes in TNBC patients (144). According to a recent study, Leptin could induce IL-18 expression in both TAMs controlled by NF-B/NF-B1 and breast cancer cells controlled by PI3K-AKT/ATF-2 signaling, which, eventually, leads to the invasion and metastasis of breast cancer cells (145).

## Future perspectives of pyroptosis in breast cancer

Since a closed association between pyroptosis and breast cancer as well as antitumor immunity, targeting pyroptosis or gene-related pyroptosis may be a treatment option for breast cancer patients and will undoubtedly become a focused area in the future. Meanwhile, with the development of bioinformatics, the association among pyroptosis, clinical outcome, and the effectiveness of immunotherapy are further comprehensively analyzed by evaluating genes and long non-coding RNAs (lncRNAs) linked to pyroptosis (146–148), which is also beneficial to discover new genes and mechanisms. However, there are only a limited number of experimental and clinical investigations that have investigated the link between pyroptosis and breast cancer. Recognizing the multidimensional roles of pyroptosis in breast cancer and applying it in cancer therapy research is still at an initial stage. In the short run, laboratories and medical institutions have an incentive to conduct studies and clinical tests with the mechanism that functions in initiating pyroptosis. Meanwhile, as a model of inflammatory death, the factors related to pyroptosis can form an inflammatory microenvironment and have a duple influence on encouraging and preventing tumor growth. The mechanism of pyroptosis and the associated factor in breast tumors must thus be further investigated in well-designed research to provide fresh therapeutic alternatives.

The study of pyroptosis is a broad and rapidly evolving field. Despite tremendous improvements in our knowledge of how pyroptosis may function in cancer and how it affects innate and adaptive immunity, several suggestions for future investigations are still worthwhile recommended. Growing evidence consistently suggested that the introduction of pyroptosis may be a valid way to treat immunity resistant cancers, which not only hold back the occurrence of the tumor but also is indispensable to anti-tumor immunotherapy (68). In that case, intensive studies are needed to develop pyroptosis based therapeutic approaches in conjunction with immunotherapy to enhance the general control of cancer. To advance our understanding of cancer research, it is important to study the effectiveness, toxicity, and adverse effects of such combinations. Moreover, further studies to explore the interplay between pyroptosis and immunotherapy agents such as ICIs will be critical when optimizing the combination therapy. And the combos and schedules in terms of time and order should be gaining prominence in preclinical testing to optimize any positive effects inside the tumor. In the end, there are still a few open questions that need ironing out. Despite the discovery of several compounds or agents that can regulate pyroptosis and present profound antitumor effects on the TME, they might not specifically target the pyroptosis pathway. More forward, one of the greatest challenges is the design of potent personalized medicine activating pyroptosis in individuals subjected to extensive safety testing. In a nutshell, the possibility of using pyroptosis as a target for anticancer modalities by galvanizing antitumor immune responses into action may develop successful therapeutic strategies.

## Conclusion

PCD is a highly regulated network that determines cell destiny and most extensively discussed subject in terms of cancer therapy. As an inflammatory PCD, pyroptosis is critical for the formation, growth, metastasis, and treatment including immunotherapy of various cancers. Despite the continuous disclosure of the molecular characteristics of the gasdermin family in pyroptosis of cancer, additional investigation into the signaling pathway, the precise mechanism of regulation, and the pathogenic prominence of pyroptosis still need innovative exploration.

Due to its diversity and medication tolerance, breast cancer has overtaken all other malignancies in terms of the number of diagnoses and is the primary cause of cancer mortality in women (1). Browsing the previous research, few works are focused on the fundamental mechanism and activity of pyroptosis in breast cancers. Various molecules and signaling pathways are implicated in gasdermins and subsequently induce pyroptosis of breast cancers (**Figure 3**). NF-κB, MEG3, JAZF1 targeted by miR-200b, JAK2/STAT3 pathway activate GSDMD while UCP1, DRD2, AMPK/SIRT1/NF-κB/BAK pathway, and STAT3/ROS/JNK pathway participate in the activation of GSDME as well as some PRRs such as AIM2, MDA5, and RIG-I can also activate GSDME. Apart from that, the complex that contains PD-L1 and STAT3 can upregulate GSDMC expression under hypoxia. *In vivo*, the current clinical testing does not allow the identification of pyroptosis. It is absolutely imperative to develop non-invasive molecular imaging approaches that can reliably identify the kind of cell death. which needs a detailed understanding of the mechanism of pyroptosis to explore feasible prognostic markers. Reduced survival is directly connected to high levels of GSDMB/C expression, which makes them a promising prognostic marker. GSDMD and GSDME, two essential pyroptosis substrates, play significant roles in the etiology and pathogenesis of breast cancer. The level of GSDME expression varies by the type of breast cancer which manifest it could be a novel premonitory marker in breast cancer while GSDMD expression is not clear fully. More research is required to reinstate GSDMB/C/E expression in breast carcinoma cells and create particular GSDMB/C/E agonists or inhibitors to make full use of it for the treatment. As for GSDMD, intensive studies on the modulation and mechanisms of the GSDMD are needed to deepen our understanding of GSDMD-mediated breast cancer and to develop GSDMD-targeting strategies that could specifically activate the pyroptosis.

More recently, increasing evidence suggested that the immunological environment in breast cancer is diverse and dynamic (149) and pyroptosis is essential for controlling the immunoreaction against breast cancer. Both pyroptosis-related inflammasomes including NLRP1, NLRP3, NLRC4, AIM2, and the following inflammatory cytokines IL-1β and IL-18 are associated with the immune system in breast cancer (**Figure 4**). Special attention required is that both activations of those inflammasomes and inflammasome-cytokines promote cancer development in certain cases, which is in line with the pyroptosis in non-canonical inflammasome pathways tend to evoke anti-tumor immunity. So selective delivery of active gasdermin proteins such as GSDMA/B/C/E *via* non-canonical inflammasome pathway in breast cancer will be another efficient strategy to antitumor. Recent studies have revealed that single chemotherapy or single immuno-oncological therapy cannot obtain an ideal therapeutic effect for breast cancer because of the immunosuppressive microenvironments. Thus, combining immunotherapy with the new treatments currently accessible has demonstrated to be quite promising. The induction of pyroptosis in breast cancer cells has generated a source for the restoration of antitumor immunity. For these reasons, the exposition on the connection between pyroptosis in non-canonical inflammasome pathways and the regulation of immune response to explore therapeutic regimens without immunosuppressive actions will provide great promise for breast cancers.

From the plenty of research accumulated above, considerable efforts are focused on the breast cancer-specific mechanisms applied for exerting pyroptosis, but there are still some problems to be solved, such as what functions other gasdermin proteins have in pyroptosis and how other factors regulate pyroptosis activation during protumor processes. Further comprehensive and better research on the function of pyroptosis in breast cancer and its crosstalk with immune therapy will be necessary.

## Author contributions

Formal analysis: CL and JW. Funding acquisition: YW. Writing–review & editing: LW, BC, and YW. All authors contributed to the article and approved the submitted version.

## Acknowledgments

This work was supported by grants from the National Natural Science Foundation of China (81902138), the Project of Public Welfare Technology Application of Zhejiang Province (LGF22H200016), and the Zhejiang Medical and Health Science and Technology Project (2022KY444).

## Conflict of interest

The authors declare that the research was conducted in the absence of any commercial or financial relationships that could be construed as a potential conflict of interest.

## Publisher’s note

All claims expressed in this article are solely those of the authors and do not necessarily represent those of their affiliated organizations, or those of the publisher, the editors and the reviewers. Any product that may be evaluated in this article, or claim that may be made by its manufacturer, is not guaranteed or endorsed by the publisher.
